# Prospective Observational Study on the Prevalence and Diagnostic Value of General Practitioners’ Gut Feelings for Cancer and Serious Diseases

**DOI:** 10.1007/s11606-021-07352-w

**Published:** 2022-01-27

**Authors:** Bernardino Oliva-Fanlo, Sebastià March, Cristina Gadea-Ruiz, Erik Stolper, Magdalena Esteva, On behalf of the CORap group

**Affiliations:** 1 Ses Roques Llises Health Centre, Majorca Department of Primary Care, Balearic Health Service (Ibsalut), Porreres, Spain; 2 APLICA, Madrid, Spain; 3 Balearic Islands Health Research Institute (IdISBa), Palma, Spain; 4 Preventive Activities and Health Promotion Research Network (RedIAPP), Barcelona, Spain; 5 Can Pastilla-Playa de Palma Health Centre, Majorca Department of Primary Care, Balearic Health Service (Ibsalut), Palma, Spain; 6 Faculty of Health, Medicine and Life Sciences, CAPHRI School for Public Health and Primary Care, Maastricht University, Maastricht, Netherlands; 7 Faculty of Medicine and Health Sciences, Department of Family Medicine and Population Health, University of Antwerp, Antwerp, Belgium; 8 Research Unit, Majorca Department of Primary Care, Ibsalut, Palma, Spain

**Keywords:** Gut feelings, Intuition, Primary care, Diagnostic reasoning, Medical problem-solving, Diagnostic validity

## Abstract

**Background:**

General practitioners (GPs) have recognized the presence of gut feelings in their diagnostic process. However, little is known about the frequency or determinants of gut feelings or the diagnostic value of gut feelings for cancer and other serious diseases.

**Objective:**

To assess the prevalence of gut feelings in general practice, examine their determinants and impact on patient management, and measure their diagnostic value for cancer and other serious diseases.

**Design:**

This prospective observational study was performed using the Gut Feelings Questionnaire (GFQ).

**Participants:**

Participants included 155 GPs and 1487 of their patients, from four Spanish provinces.

**Main Measures:**

Sociodemographic data from patients and GPs; the reasoning style of GPs; the characteristics of the consultation; the presence and kind of gut feeling; the patient’s subsequent contacts with the health system; and new cancer and serious disease diagnoses reported at 2 and 6 months post-consultation.

**Key Results:**

GPs experienced a gut feeling during 97% of the consultations: a sense of reassurance in 75% of consultations and a sense of alarm in 22% of consultations. A sense of alarm was felt at higher frequency given an older patient, the presence of at least one cancer-associated symptom, or a non-urban setting. GPs took diagnostic action more frequently after a sense of alarm. After 2 months, the sense of alarm had a sensitivity of 59% for cancer and other serious diseases (95% CI 47–71), a specificity of 79% (95% CI 77–82), a positive predictive value of 12% (95% CI 9–16), and a negative predictive value of 98% (95% CI 86–98).

**Conclusions:**

Gut feelings are consistently present in primary care medicine, and they play a substantial role in a GP’s clinical reasoning and timely diagnosis of serious disease. The sense of alarm must be taken seriously and used to support diagnostic evaluation in patients with a new reason for encounter.

## BACKGROUND

Uncertainty around diagnosis is one of the biggest challenges that a clinician faces when caring for a patient. This is particularly relevant for general practitioners (GPs)^1^, whose work is associated with one of the highest perceptions of uncertainty^2^. GPs are confronted with an immense range of symptoms, and in some cases a seemingly minor symptom can indicate a serious diagnosis^3^. For example, most lower-back pain disappears within a year; however, in 1 of 350 patients with backache, the pain will be the guiding sign for a serious diagnosis^4^. This uncertainty forces GPs to optimize the use of their analytical and non-analytical reasoning tools. In this sense, the use of intuition in medicine has long been recognized as part of the *art of medicine* and even as representing *tacit knowledge essential to good practice*^5,6^*.* Intuitive sensations, called “gut feelings,” have been described as a “useful light that goes on suddenly to announce that there is something unusual.”^7^ GPs have been reported to recognize the existence of gut feelings and consider them a useful tool for decision-making^8,9^, and even a separate track in their clinical reasoning^10^.There are two kinds of gut feelings: a *sense of alarm* that leads a GP to worry about a patient’s health status even if they have not yet found any specific indication; and a *sense of reassurance* that leads a GP to feel confident about the patient’s management and outcome even though they may not be certain about the diagnosis, a sense that everything fits in^8^.

There are many studies regarding the use of gut feelings by GPs, hospital specialists, and nurses^9,11–17^. GPs reported using their gut feelings in suspecting cancer^9,17–20^and other serious diseases^21,22^. In Denmark and the UK, the GPs’ sense of alarm has been accepted as a valid reason for referring a patient to specific pathways of cancer diagnosis^23,24^. It has been suggested that gut feelings’ diagnostic values are routine GP’s consultations, where serious diseases and cancer have a low prevalence^25^. However, few studies have evaluated the frequency and diagnostic value of gut feelings in primary care consultations^20^. Hjertholm et al. found that the suspicion of cancer and serious disease in primary care consultations had a prevalence of 5.7% and a positive predictive value (PPV) of 9.8%^26^. Donker et al. observed a PPV of 35% for cancer-related gut feelings, and reported that this value increased according to the ages of the patient and the doctor^12^. In order to objectively measure gut feelings, a Gut Feeling Questionnaire (GFQ) was created and validated in a Dutch context^22^; since then, it has been made available in seven languages^27,28^. The GFQ determines whether a gut feeling has arisen during a consultation. In a study using the GFQ, Barais et al. found that GPs had a gut feeling in 99.16% of consultations concerning patients with dyspnea or chest pain, corresponding to a sense of alarm in 35% of these cases and a sense of reassurance in 65% of the cases. Among patients with dyspnea or chest pain, the presence of a sense of alarm increased the probability of a life-threatening disease from 20 to 35%, while the presence of a sense of reassurance decreased the probability to 12%^13^.

Non-analytical, intuitive reasoning is a substantial part of the diagnostic process; it induces and guides analytical reasoning and deliberate action. However, the prevalence, diagnostic value, and determinants of gut feelings are not yet fully known. More knowledge of these aspects might lead to a better understanding of the consultation process and help practitioners undertake timely diagnostic evaluation and avoid errors.

This study aimed to assess the prevalence and determinants of gut feelings in general practice, the subsequent management of patients in light of the kind of gut feeling experienced by the GP, and the diagnostic value of gut feelings for cancer and other serious diseases.

## METHODS

In this prospective observational study, we used the Spanish and Catalan versions of the GFQ. The work was carried out in primary care centers of four Spanish provinces (Balearic Islands, Madrid, Barcelona, and Lugo) during 2019–2020. Participants were GPs and their patients. The protocol of the study has already been published^29^.

### Participants

GPs were invited to participate during workshops held in the health centers. Those who accepted the invitation were instructed on data gathering. During at least one working day, GPs included consecutive patients with at least one new reason for consultation. Exclusion criteria were consultations with non-residents, terminally ill patients, or patients younger than 18 years old, and consultations for bureaucratic reasons. At the end of each consultation (index consultation), patients were given oral and written information about the study and signed an informed consent document.

### Measurements

We collected sociodemographic and practice data on the participating GPs (age, sex, training tasks, rural/non-rural health center, and years with the same list of patients). We used a 4-item Likert scale validated by Martínez-Cañabate et al.^30^ in her PhD thesis. Each item has 4 possible answers, from completely disagree to completely agree. The scale assesses whether the professional carries out a practice more oriented to the biological (lower scores) or psychosocial (higher scores) sphere^31,32^. GPs completed the Rational-Experiential Inventory (REI)^33^. This Likert scale has 40 items and has been validated in Spanish population. Each item has 5 possible answers, from completely disagree to completely agree. The REI measures rational and experiential thinking styles and includes subscales of self-reported ability and engagement with each thinking style^34^.

After each index consultation, GPs recorded sociodemographic data obtained from the patient and how long they had been on the doctor’s list. Data about the visit were recorded, such as the type and consultation duration (longer or shorter than 6 min), the language used, and the presence of cancer-associated symptom(s)^35–38^(Table 1). Finally, the GP completed a printed Spanish or Catalan version of the 11-item GFQ^9,28,39^. Item 1 (repeated at the end as item 11) assesses whether the patient’s case elicited a gut feeling in the consultation. Items 2–6 are rated using a 5-point Likert scale that ranges from completely disagree to completely agree. Item 2 concerns the sense of reassurance and items 3–6 relate to the sense of alarm. A sense of alarm is considered present when the answer to item 1 or 11 indicates a sense of alarm, or when the answer to item 1 or 11 is “not applicable” and at least one of the scores of items 3–6 is higher than 3. A sense of reassurance is considered present when the answer to item 1 or 11 indicates a sense of reassurance or when the answer for item 1 or 11 is “not applicable” and the score for item 2 is higher than 3. A gut feeling is considered to be absent when the answers for items 1 and 11 are both “not applicable,” none of the scores for items 3–6 is higher than 3/4, and the score for item 2 is lower than 4/5.

**Table 1:** Signs and Symptoms Associated with a Higher Predictive Value for Cancer^19,20,28,29^

- Unintentional weight loss - Anemia - Anorexia - Asthenia - Altered bowel habits: ○ Diarrhea ○ Constipation - Persistent dyspepsia - Dysphagia - Cough - Dysphonia - Lower urinary tract symptoms - Unusual bleeding: ○ Hemoptysis ○ Hematuria ○ Rectal bleeding ○ Vaginal postmenopausal bleeding - Breast lump - Abdominal mass - Unusual pain	

Two months and 6 months after the index consultation, we reviewed primary care and hospital clinical records to collect new diagnoses of cancer (except non-melanoma skin cancer) and other serious diseases among the participating patients. Recurrence of cancer in patients considered disease-free at the time of the index consultation was regarded as a new diagnosis. Beginning with the list of serious diseases published by Hjertholm et al.^26^, two researchers independently judged whether a newly diagnosed disease was “serious” or not. When there was disagreement, a third researcher made the final decision. Six months after the index consultation, we also recorded patient contacts with health care services.

### Statistical Analysis

We performed a descriptive analysis of all selected variables to describe sample characteristics and the prevalence of gut feelings. A bivariate analysis was carried out, in which the presence of a sense of reassurance or alarm was compared with the characteristics of the GP, patient, and consultation. We used the chi-square test for categorical variables, and Student’s *T*-test for continuous variables. OR and 95% CI were calculated. A multivariate logistic regression analysis was done to assess the independent relationships between the variables and the kind of gut feeling. Variables with *p*≤ 0.20 were introduced in the model^40^. We assessed changes in the coefficients at each step to detect confusion, and tested interactions. Sensitivity, specificity, positive and negative predictive values (PPVs, NPVs), and positive and negative likelihood ratios (LR+, LR−) were calculated for the sense of alarm and the sense of reassurance. We assumed that the sense of alarm aims to identify patients with high probability for a serious outcome, while the sense of reassurance aims to identify patients with low probability for a serious outcome. Logistic multivariate analysis was also used to calculate the risk of serious disease depending on the type of gut feeling, adjusted for patient age, sex, visit type, visit duration, and cancer-related symptom(s). We assessed goodness of fit for every model with the Hosmer-Lemeshow test. Analysis was done with SPSS.v.25.

## RESULTS

**Figure 1:**
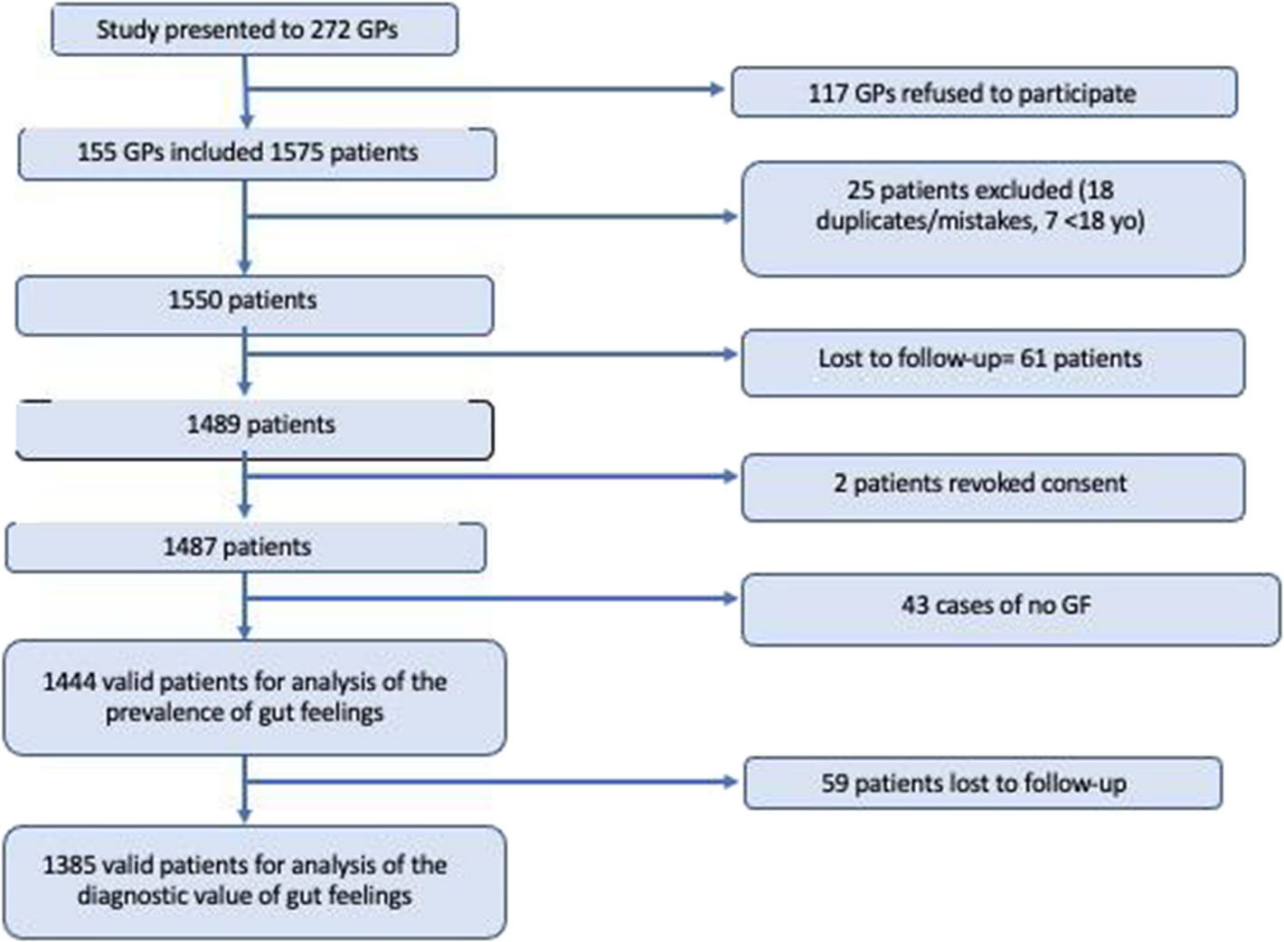
Flowchart of general practitioners and patients included.

We invited 272 GPs; of them, 155 participated. The GPs reported on 1487 patients (63.2% female) over 328 working days (see flowchart in Figure 1). Most of the patients were Spanish-born and lived in urban environments; their mean age was 51.9 years. Nearly six of 10 patients presented at least one cancer-associated symptom. The characteristics of the GPs, patients, and consultations are described in Table 2.

**Table 2:** Characteristics of General Practitioners, Patients, and Index Consultations

General practitioners		*N* (%)
Sex	Female Male	109 (70.3) 46 (29.6)
Language	Spanish Catalan Other	112 (72.2) 42 (27.1% 1 (0.6)
Environment	Urban Extra-urban	134 (86.4) 21 (13.5)
GP trainer	Yes No	63 (40.6) 92 (59.3)
Age, *mean (SD)*		46.1 (9.67)
Years same list, *mean (SD)*		7.8 (7.28)
Working days included, *mean (SD)*		2.1 (1.25)
Patients included by each GP, *mean (SD)*		9.5 (5.37)
NFC engagement, *mean (SD)*		3.6 (0.51)
NFC ability, *mean (SD)*		3.5 (0.4)
FI engagement, *mean (SD)*		3.2 (0.4)
FI ability, *mean, (SD)*		3.3(0.5)
Martínez-Cañabate, *mean (SD)*		9.7 (2.4)
Patients		***N*(%)**
Sex	Female Male Unknown	911 (61.2) 530 (35.6) 46 (3.0)
Age, *mean (SD)*		51.9 (19.2)
Country of origin	Spain Other Unknown	1096 (75.1) 362 (24.8) 29
Patient language	Spanish Catalan Other Unknown	1118 (75.4) 237 (16.0) 126 (8.5) 6
Environment	Urban Extra-urban	1268 (85.2) 219 (14.7)
Prior knowledge	Yes No	1056 (74.4) 363 (25.6)
Number years of GP-patient prior knowledge, *mean (SD)*		4.78 (5.7)
Symptoms of possible cancer	No ≥1	595 (40.0) 892 (59.9)
Index consultations		***N*%**
Same language patient-GP during consultation	Yes No Unknown	1086 (74.2) 387 (25.7) 14
Same sex patient-GP	Yes No Unknown	778 (55.8) 611 (44.1) 98
Length of consultation >6 min	Yes No Unknown	991 (70.1) 421 (29.8) 75
Gut feeling	Sense of reassurance Sense of alarm Inconclusive	1120 (75.3) 324 (21.7) 43 (2.8)
Type of visit	Scheduled Non-scheduled	1145 (77.0) 342 (23.0)
Patients visited, *mean (SD)*		26.44 (8.04)

*GP* general practitioner, *SD* standard deviation, *NFC* need for cognition, *FI* faith in intuition

### Prevalence of Gut Feelings

GPs experienced a gut feeling during 97.1% of the consultations: a sense of reassurance was recorded in 1120 consultations (75.3%) and a sense of alarm was recorded in 324 consultations (21.7%). In 43 consultations, the GFQ did not determine a gut feeling. These cases were excluded from the analysis. The GPs, patients, and consultations characteristics are categorized by the type of GF present in Table 3.

**Table 3:** Relationship of General Practitioners, Patients and Consultation Characteristics, and Type of Gut Feeling

Variables	Global *N* (%)	SA *N* (%)	SR *N* (%)	OR SA/SR (CI 95%) (non-adjusted)	*P*	OR SA/SR (CI 95%) (adjusted model)	*P*
Total	1444	324 (22.4)	1120 (77.6)			–	
GP characteristics							
Sex (GP)							
Female	1005 (69.6)	237 (23.6)	768 (76.4)	1			
Male	439 (30.4)	87 (19.8)	352 (80.2)	0.80 (0.6–1.06)	0.11	–	–
Environment							
Non-urban	216 (14.9)	68 (31.4)	148 (68.5)	1		1	
Urban	1228 (85.04)	256 (20.8)	972 (79.1)	0.50 (0.40– 0.70)	0.001	1.57 (1.09–2.25)	0.015
GP trainer							
Yes	606 (41.9)	146 (24.0)	460 (75.9)	1			
No	838 (58.0)	178 (21.2)	660 (78.7)	0.85 (0.60–1.10)	0.20	–	–
Age (GP)							
Mean (SD)	46.0 (9.6)	46.43 (9.8)	46.03 (9.5)	1.04 (0.99–1.01)	0.51	–	–
Years same list							
*Mean (SD)*	7.95 (7.36)	7.74 (6.79)	8.09 (7.56)	0.99 (0.97–1.01)	0.45	–	–
GP’s NFC engagement							
Mean (SD)	3.6 (0.48	3.7 (0.4)	3.6 (0.4)	1.58 (1.21–2.06)	0.001	1.68 (1.25–2.27)	0.001
GP’s NFC ability							
Mean (SD)	3.5 (0.4)	3.5 (0.4)	3.5 (0.4)	1.18 (0.88–1.57)	0.25	–	–
GP’s FI engagement							
Mean (SD)	3.2 (0.4)	3.3 (0.4)	3.2 (0.4)	1.13 (0.85–1.51)	0.37	–	–
GP’s FI ability							
Mean (SD)	3.4 (0.7)	3.4 (0.6)	3.4 (0.8)	0.98 (0.83–1.16)	0.88	–	–
Martínez-Cañabate Scale							
Mean (SD)	9.4 (2.4)	9.3 (2.4)	9.4 (2.4)	0.98 (0.93–1.03)	0.50	–	–
Patient characteristics							
Sex (patient)							
Female	887 (63.4)	199 (22.4)	688 (77.6)	1			
Male	512 (36.6)	111 (21.7)	401 (78.3)	0.9 (0.7–1.2)	0.74	–	–
Age (patient)							
Mean (SD)	51.9 (19.2)	55.3 (19.6)	51.0 (19.0)	1.01 (1.005–1.018)	0.001	1.01 (1.03–1.02)	0.004
Country of origin							
Spain	1069 (75.5)	238 (22.3)	831 (77.7)	1			
Other	347 (24.5)	77 (22.2)	270 (77.8)	0.9 (0.7–1.3)	0.97	–	–
Prior knowledge							
No	363 (25.6)	81 (22.3)	282 (77.7)	1			
Yes	1056 (74.4)	235 (22.3)	821 (77.7)	1.003 (0.7–1.3)	0.98	–	–
Years of prior knowledge							
*Mean (SD)*	4.7 (5.7)	4.5 (5.3)	4.9 (5.9)	0.99 (0.96–1.01)	0.38	–	–
Cancer=related symptoms							
No	582 (40.3)	94 (16.2)	488 (83.8)	1		1	
≥1	862 (59.7)	230 (26.7)	632 (73.3)	1.8 (1.4–2.4)	<0.001	1.83 (1.36–2.46)	<0.001
Consultation characteristics							
Language used (GPs)							
No	374 (26.2)	112 (29.9)	262 (70.1)	1		1	
Yes	1056 (73.8)	208 (19.7)	848 (80.3)	0.5 (0.4–0.7)	<0.001	1.62 (1.20–2.18)	<0.001
Same sex patient-GP							
No	620 (44.3)	137 (22.1)	483 (77.9)	1			
Yes	778 (55.7)	173 (22.2)	605 (77.8)	1.01 (0.7–1.3)	0.95	–	–
Length of consultation >6’							
No	410 (29.9)	42 (10.2)	368 (89.8)	1		1	
Yes	959 (70.1)	260 (27.1)	699 (72.9)	3.2 (2.2–4.6)	<0.001	2.76 (1.92–3.97)	<0.001
Type of visit							
Scheduled	1111 (76.9)	252 (22.7)	859 (77.3)	1			
Rest of visits	333 (23.1)	72 (21.6)	261 (78.4)	0.9 (0.6–1.2)	0.68	–	–
Patients visited							
*Mean (SD)*	26.4 (8.05)	25.4 (8.2)	26.7 (7.8)	0.97 (0.96–0.99)	0.01	–	–

*GPs* general practitioners, *SA* sense of alarm, *SR* sense of reassurance, *No GF* no gut feelings detected, *CI* confidence interval, *SD* standard deviation

*R*^2^Nagelkerke= 0.114; Hosmer and Lemeshow goodness of fit test = 0.114

We found no difference in the frequency of reassurance or alarm regarding most of the determinants studied. The sense of alarm was more prevalent, and the sense of reassurance was less prevalent under the following conditions: when GPs’ NFC engagement scores (rational reasoning) were higher; in consultations with older patients; when a patient presented at least one cancer-associated symptom; in non-urban areas; or when the language used during the consultation was not the GP’s native language. Regarding the features of the consultations, the prevalence for a sense of alarm was higher in consultations that lasted longer than 6 min or with fewer patients seen that day. Our multivariate analysis (see Table 3) confirmed the above-described results found in the bivariate analysis, except for the number of patients visited in the same day.

### Actions During Follow-up

Table 4 shows the actions taken by GPs during the 6 months after the index consultation, categorized by the kind of gut feeling. Patients visited their GP more frequently after GPs experienced a sense of alarm than a sense of reassurance. GPs more frequently ordered laboratory tests, radiological investigations, and primary care procedures after experiencing a sense of alarm and referred more frequently to both outpatient services and the emergency department. There was no difference in patients’ sick leave based on the type of feeling experienced by the GP.

**Table 4:** Actions Taken During the Subsequent 6 months

	SA *N*=324	SR *N*=1120	*P*
Patients visiting a GP (%) *Mean (SD)*	310 (95.7) 4.8 (4.1)	960 (85.7) 3.56 (3.6)	<0.001 <0.001
Patients with laboratory tests (%) *Mean (SD)*	200 (61.7) 0.8 (0.9)	463 (41.3%) 0.5 (0.7)	<0.001 <0.001
Patients with radiology tests (%) *Mean (SD)*	99 (30.6) 0.3 (0.6)	226 (20.2) 0.19 (0.4)	<0.001 <0.001
Patients referred to outpatients services (%) *Mean (SD)*	169 (52.2) 0.65 (0.7)	361 (32.2) 0.3 (0.6)	<0.001 <0.001
Patients referred to ED (%) *Mean (SD)*	82 (25.3) 0.2 (0.4)	115 (10.3) 0.08 (0.3)	<0.001 <0.001
Patients with primary care procedures (%) *Mean (SD)*	128 (39.5) 0.6 (1.1)	317 (28.3) 0.4 (1.01)	<0.001 0.002
Patients with sick leaves (%) *Mean (SD)*	148 (43.8) 0.2 (0.5)	452 (40.4) 0.2 (0.6)	0.26 0.44

*SA* sense of alarm, *SR* sense of reassurance, *No GF* no gut feelings detected, *CI* confidence interval, *SD* standard deviation

### Diagnostic Value

The presence of a diagnosis of cancer or serious disease could be evaluated in 1385 patients (see Figure 1). At 2 months after the index consultation, 64 patients (4.6%) had been newly diagnosed with cancer or another serious disease. At 6 months, a total of 116 patients had been newly diagnosed with a serious disease (8.3%; nine with cancer).

Diagnostic values are shown in Table 5. After 2 months, the sense of alarm for cancer or a serious disease had a sensitivity of 59.3%, specificity of 79.4%, a PPV of 12.2%, an NPV of 97.5%; an LR+ of 2.8, and an LR− of 0.5. After 6 months, most of these figures were similar for the sense of alarm, except that the PPV was 18.3% and the NPV was 94.5%.

**Table 5:** Diagnostic Value Parameters of Gut Feelings for Cancer and Serious Disease and Risk of Cancer and Serious Disease Depending on the Type of Gut Feeling (*N*=1385)

Time after consultation	Sensitivity % (95% CI)	Specificity % (95% CI)	PPV % (95% CI)	NPV % (95% CI)	LR+ (95% CI)	LR- (95% CI)	Accuracy (95% CI)	Non-adjusted OR (95% CI)	Adjusted OR (95% CI)
2 months after SA	59.3 (47.1–70.5)	79.4 (77.1–81.5)	12.2 (9.06–16.3)	97.5 (86.4–98.3)	2.8 (2.7–3.0)	0.5 (0.4–0.5)	78.4 (76.2–80.5)	5.63 (3.36–9.44)	5.3* (3.09–9.08)
6 months after SA	49.14 (40.2–58.1)	80.1 (77.7–82.1)	18.3 (14.4–23.1)	94.5 (92.9–95.7)	2.4 (2.3–2.5)	0.6 (0.61–0.66)	77.4 (75.2–79.6)	3.88 (2.62–5.72)	3.67** (2.42–5.56)
2 months after SR	79.4 (77.1–81.5)	59.3 (47.1–70.5)	97.5 (6.4–98.3)	12.2 (9.06–16.3)	1.9 (1.8–2.1)	0.3 (0.33–0.36)	78.4 (76.2–80.5)	0.17 (0.1–0.29)	0.19*** (0.1–0.33)
6 months after SR	80.1 (77.7–82.1)	49.14 (40.2–58.1)	94.5 (92.9–95.7)	18.3 (14.4–23.1)	1.57 (1.5–1.6)	0.4 (0.390.42)	77.4 (75.2–79.6)	0.25 (0.17–0.38)	0.27**** (0.17–0.41)

*PPV* positive predictive value, *NPV* negative predictive value, *LR+* positive likelihood ratio, *LR−* negative likelihood ratio

*Hosmer-Lemeshow test=0.22; **Hosmer-Lemeshow test=0.53; ***Hosmer-Lemeshow test=0.19; ****Hosmer-Lemeshow test=0.79

The adjusted OR for a serious diagnosis after 2 months was 5.3 after a sense of alarm and 0.19 after a sense of reassurance. Six months after the index consultation, the adjusted OR was 3.6 after a sense of alarm and 0.2 after a sense of reassurance.

## DISCUSSION

### Summary of Findings

This is the first study seeking to estimate the prevalence and diagnostic value of gut feelings in the consultations of GPs. Our study showed that GPs had a gut feeling almost every time they consulted with a patient for a new reason; these feelings were a sense of reassurance approximately 75% of the time. A more frequent sense of alarm was associated with various determinants, such as the GP being more engaged with analytical reasoning, the patient’s age, the practice being located in a non-urban area, the presence of at least one cancer-associated symptom, and incongruence in the native languages of the patient and GP. We also observed that the presence of a sense of alarm increased the number of tests performed and the referrals to secondary care for further investigation. The sense of alarm experienced by the GP increased the possibility that the patient would receive a new diagnosis of cancer or another serious disease by 2 months (adjusted OR 5.3) and 6 months (adjusted OR 3.6) after the initial consultation. This possibility decreased after the GP’s perception of a sense of reassurance, with an adjusted OR of 0.19 at 2 months and 0.27 at 6 months. The presence of a sense of alarm increased the likelihood of the diagnosis of a cancer or a serious disease at 2 months from the consultation from 4.6 to 12.3% and from 8.4 to 18.4% at 6 months, while the presence of a sense of reassurance decreased these likelihoods to 2.4% and 5.9%, respectively.

### Strengths and Limitations

The GFQ is a validated measure for determining gut feelings. Our prospective design enabled us to obtain accurate and reliable results. The use of primary care and hospital electronic clinical records prevented loss of information, such as unrecorded diagnoses.

We did not reach the estimated sample size of consultations^29^, as 43% of the GPs decided not to participate. Therefore, our data lacked the power needed for us to draw conclusions about the diagnostic value of GFs related exclusively to cancer. The distributions of participant GPs by age, sex, and non-urban vs. urban environment were essentially the same as those previously observed among Spanish GPs^41,42^.

The outbreak of the COVID-19 pandemic during the last months of data collection might have influenced our results. All the index consultations occurred prior to the pandemic, but the follow-up periods for 1/3 of the cases ended after the pandemic started. Thus, although COVID-19 was not a diagnostic possibility during the index consultation, patients were exposed to the new disease during the follow-up period. Moreover, the Spanish National Health System stopped all non-urgent activity during the first months of the pandemic, probably delaying some cancer diagnoses^43,44^. We considered COVID-19 to be a serious disease when patients suffered complications, needed hospitalization, or died. We found 36 confirmed COVID-19 cases among participants, including 35 mild cases and 1 with pneumonia. Otherwise, the prevalence of cancer and the other serious diseases was comparable between the present study and previous relevant reports^26^.

### Comparison with Existing Literature

The consistent appearance of gut feelings during the consultations indicates that GPs habitually use intuitive reasoning. The intuitive decision-making system is fast, automatic, effortless, and difficult to control^45,46^. In primary care health centers, which are characterized by massive numbers of consultations and strict time constraints, the contribution of “intuition” to the decision-making process is obvious. The high prevalence of gut feelings involving a sense of reassurance is in line with the low probability of serious disease in primary care, as many complaints are innocent and temporary indispositions^47^.

Studies measuring gut feelings with non-validated tools found a much lower prevalence of the sense of alarm^26,37,48^. The Hawthorne effect^49^, which is how the awareness of being studied may impact the behavior of the study subjects^50^, should be considered a possible source of bias. Although the GPs did not know if their answers to the GFQ would reflect a sense of alarm or a sense of reassurance, they might have changed their behavior and been more suspicious when interpreting the patient’s symptoms during their participation in the study, potentially leading to an overestimation of the sense of alarm.

If the high prevalence of the sense of alarm found in our study using the GFQ was an overestimation, then it had inevitably influenced the predictive value. Barais et al.^13^ used the GFQ among French GPs in patients consulting for dyspnea or chest pain; the authors observed that gut feelings were present in 99.15% of consultations, with 35% of them representing a sense of alarm and 65% representing a sense of reassurance. The higher prevalence of a sense of alarm, compared to that found our study, can be easily explained because these authors selected patients with dyspnea and chest pain, who have a much higher risk of serious outcome. We assume that the Hawthorne effect is at least partially responsible for our finding that a high proportion of the gut feelings experienced by GPs corresponded to a sense of alarm. To mitigate this effect, several strategies have been proposed that should be considered in future research on gut feelings, such as assuring the participants that the objective of the study is to identify gut feelings without judging the clinician’s performance, triangulating the collection of information, and collecting information over long periods of time while discarding the first set of data collected^51^. Another less probable explanation could be that case vignettes from real practice were used to validate the GFQ and perhaps the cut-off values need to be refined in the context of real consultations^39^.

Our results showed that the kind of gut feelings was influenced by some characteristics of the GPs, patients, and/or of the consultations. The style of reasoning (rational or intuitive) did not appear to generally affect the occurrence of gut feelings. However, somewhat to our surprise, GPs prone to rational reasoning had more frequent experiences of a sense of alarm. The Spanish non-urban population is older than the urban population, which could explain the higher prevalence of GPs having a sense of alarm in non-urban areas^42^. The presence of at least one cancer-associated symptom increased the prevalence of a sense of alarm, which is consistent with previous published evidence^36,37^. The sense of alarm may activate the diagnostic process by stimulating a GP to formulate and weigh working hypotheses involving a serious outcome^11^ that is, the sense of alarm was associated with a longer consultation.

Doctors of different specialties have acknowledged the presence of gut feelings in their diagnostic process^14,15,21^, although they considered that it is more frequent and appropriate among GPs because of the greater number of diagnostic possibilities a GP faces after a patient. Intuition played a greater role and was more widely accepted in specialties like general internal medicine, pediatrics, and psychiatry^15^. These specialties, along with family medicine, are the ones where physicians have a higher perception of uncertainty in their daily work^2^.

We found an increasing number of GP visits, tests, and referrals for patients with whom the GP experienced a sense of alarm. Our results are comparable with those observed by Hjertholm et al.^26^, where the number of GP consultations, primary-care specialist, and diagnostic imaging increased in the 2-month period after a consultation when the GP had a suspicion of serious disease, while the use of hospital services (inpatient and outpatient increased both 2 and 6 months after). These findings could be expected, as this gut feeling induces the diagnostic process of gathering more data.

Regarding the diagnostic value of gut feelings, other authors also observed increased probabilities of serious disease after a sense of alarm. Hjertholm et al.^26^ found that the risk of a diagnosis of cancer or another serious disease was 2.98 higher 2 months after the index consultation in the case of an sense of alarm. Ingeman et al.^52^ found that 24% of patients with whom the GP felt a gut feeling of cancer were finally diagnosed with cancer. A meta-analysis on the diagnostic utility of gut feelings in diagnosing cancer in primary care showed that a gut feeling associated with cancer increased the odds of cancer four times^20^. These results justified the decision made in Denmark and the UK to accept the GPs’ gut feeling as a valid reason for referring a patient to specific pathways of cancer diagnosis^23,24^.

The value of LR+ of a sense of reassurance (1.95) implies that the pretest probability of serious disease decreases from 8.4 to 5.5%, and thus did not contribute greatly to ruling out cancer or a serious disease, so still the GP has to consider several hypotheses before discarding a serious diagnosis. The LR+ of a sense of alarm (2.8) modified the pretest probability for cancer or serious disease from 8.4 to 18.4%. As proposed by Barais^51^, given the very low prevalence of serious diseases, LR+ values between 2 and 5 could be of interest since they increase the probability of serious disease by 15–30%. Therefore, a sense of alarm should be taken seriously in general practice, and clinicians should follow up patients with an analytical reasoning track.

## Conclusions

The results of our study showed that gut feelings are substantially present in primary care. Gut feelings, especially a sense of alarm, contribute valuably to the diagnostic process and must be taken seriously when seeing patients with a new reason for encounter, which should lead to a diagnostic review. A gut feeling is a substantial part of clinical reasoning and supports GPs in timely diagnosing cancer or other serious diseases. Rational reasoning-prone GPs did not differ from their intuitive reasoning-prone colleagues with respect to experiencing gut feelings. Medical students must be trained in becoming aware of their own gut feelings and how to deal with them. Further research should focus on the significance of gut feelings related to specific symptoms and signs, and on the factors that could increase the prognostic and diagnostic value of GPs’ gut feelings.

## Data Availability

The datasets used and/or analyzed during the current study are available from the corresponding author on reasonable request.

## **Abbreviations**

GP: General practitioner
GF: Gut feeling
GFQ: Gut Feelings Questionnaire
REI: Rational Emotional Inventory
NFC: Need for cognition
FI: Faith in intuition
EKG: Electrocardiogram
OR: Odds ratio
CI: Confidence interval
LR: Likelihood ratio
PPV: Positive predictive value
NPV: Negative predictive value
SD: Standard deviation
